# The effect on clinical outcomes when targeting spinal manipulation at stiffness or pain sensitivity: a randomized trial

**DOI:** 10.1038/s41598-020-71557-y

**Published:** 2020-09-03

**Authors:** Casper Glissmann Nim, Gregory Neil Kawchuk, Berit Schiøttz-Christensen, Søren O’Neill

**Affiliations:** 1 Medical Research Unit, Spine Centre of Southern Denmark, University Hospital of Southern Denmark, Odense, Denmark; 2 Department of Physical Therapy, University of Alberta, Edmonton, Canada; 3 Department of Regional Health Research, University of Southern Denmark, Odense, Denmark

**Keywords:** Randomized controlled trials, Musculoskeletal system

## Abstract

The mechanisms underlying pain relief following spinal manipulative therapy (SMT) are not understood fully although biomechanical and neurophysiological processes have been proposed. As such, we designed this randomized trial to elucidate the contributions of biomechanical and neurophysiological processes. A total of 132 participants with low back pain were randomly assigned to receive SMT at either the lumbar segment measured as the stiffest or the segment measured as having the lowest pain threshold. The primary outcome was patient reported low back pain intensity following treatment. Secondary outcomes were biomechanical stiffness and neurophysiological pressure pain threshold. All outcomes were measured at baseline, after the fourth and final session and at 2-weeks follow-up. Data were analyzed using linear mixed models, and demonstrated that the SMT application site did not influence patient reported low back pain intensity or stiffness. However, a large and significant difference in pressure pain threshold was observed between groups. This study provides support that SMT impacts neurophysiological parameters through a segment-dependent neurological reflex pathway, although this do not seem to be a proxy for improvement. This study was limited by the assumption that the applied treatment was sufficient to impact the primary outcome.

## Introduction

### Treatment of low back pain

Low back pain (LBP) is now the number one cause for years lived disability worldwide^1^. In most cases, a specific pathoanatomical cause of LBP cannot be identified^2^. Without a specific therapeutic target, a predictably large and diverse spectrum of interventions are available to clinicians that range from joint mobilization to spinal fusion surgery^3^. Given these almost endless possibilities, clinical guidelines rate education and exercise as first line therapy for low back pain often in combination with manual therapy^3^. Although, these guideline recommendations are generally clear and unambiguous, it is challenging for clinicians to implement them in practice (e.g. which exercises to recommend, how often, and which patients to offer manual therapy etc.).

### Spinal manipulative therapy

Spinal manipulative therapy (SMT) is a manual therapy recommended as a second line intervention for LBP in most clinical guidelines^4^. However, like other conservative treatments, there is little evidence or consensus regarding the specifics of SMT application such as which patients are likely to respond, which type of SMT should be used, and which dose/frequency of SMT is optimal.

While the specific SMT technique does not seem to be important^5–7^, there are at least two theoretical rationales for where to apply SMT: at the site of greatest biomechanical dysfunction or the site of greatest pain sensitivity. As the goal of SMT is to restore normal function to segments with biomechanical dysfunctions^8^, it may be surprising to some that the evidence for identifying such dysfunction is sparse. A narrative review reported that clinicians use a variety of different ways to determine the application site of SMT, often consisting of palpation using patient reported pain when provoking specific segments, and a subjective assessment of lumbar stiffness^9^.

### Biomechanical dysfunction as the site for SMT application

Spinal palpation used to identify biomechanical dysfunction in the spine has been thoroughly researched. While its intra-observer reproducibility is acceptable, the inter-observer reliability is, not surprisingly, less convincing^10^. Despite these conflicting results, palpating to identify hypomobile segments is included in a clinical prediction rule, and part of standard clinical examination prior to SMT^11^. As there is no universal agreement on the characteristics of a segmental dysfunction it is difficult to identify, quantify and thereby measure changes in these properties. While these manual methods of identifying dysfunction are problematic, a preliminary study using instrumentation found that patients with LBP who respond to SMT with improvements in self-reported disability had associated decreases in post-SMT stiffness^12^. The study by Wong et al.^12^ measured stiffness at L3 with a single mechanical indentation^13^. A more recent version of this device, the VerteTrack (VT), has recently been developed. The VT has the ability to approximate mechanical indentation over a large spinal region, and, thereby, obtain a rapid measure of lumbar stiffness at each lumbar segment^14^. This potentially aids in quantifying segmental stiffness, and in turn, is thought to direct the application of SMT more accurately.

### Lumbar pain sensitivity as the site for SMT application

Spinal manipulative therapy appears to have a hypoalgesic effect on pain sensitivity, both in the region where SMT is applied and more widespread^15^. This difference in pain sensitivity appears to manifest in both patients with musculoskeletal conditions^16^ and healthy individuals alike^17^, suggesting a short-term neurophysiological effect of SMT on pain sensitivity, irrespective of underlying pain conditions. In recent decades, pain sensitivity has been quantified reliably using quantitative sensory testing (QST)^18^. Pressure pain threshold (PPT) can quantify the amount of pressure needed to induce a perception of subjective pain^19, 20^, and be used to gain insight in local mechanical pain sensitivity at each lumbar segment. The authors, have no knowledge of any study using PPT to determine the site for SMT. This could potentially aid in identifying specific segmental pain sensitivity.

### Study rationale

The clinical effect of SMT for persistent LBP (current LBP for more than 3 months) is comparable to other guideline recommended conservative treatments (e.g. exercise, education etc.) with regards to pain and disability^21^. However, the underlying mechanisms of the clinical effects of SMT remains unclear. A normalization of both segmental biomechanics and pain sensitivity could arguably be explanations for pain relief, and it is not known whether the effects of SMT can be improved by targeting spinal segments characterized by parameters such as segmental stiffness and pain sensitivity.

In this study, patients with persistent low back pain were enrolled. For each participant, the most stiff and most painful vertebral segments were identified. Spinal manipulative therapy was then provided at the same segment over four sessions. The segment was determined accordingly to the baseline randomization as either the most stiff or most painful segment.

### Objectives

The primary aim of this study is to examine if spinal manipulation is more effective in regards to reducing patient reported low back pain intensity when directed at spinal segmental stiffness or segmental pain sensitivity in a cohort of persistent low back pain patients. The secondary aims were to measure between group mean changes in (i) lumbar stiffness and (ii) pressure pain threshold.

## Methods

This trial was approved by the Regional Committees on Health Research Ethics for Southern Denmark (ID: S-20160201) and the Danish Data Protection Agency. The trial was registered at ClinicalTrials.gov 11/09/2019—identifier: NCT04086667. All participants provided informed, written and oral consent before entering the study. The project was conducted in accordance with the Helsinki-II declaration. The trial is reported according to the CONSORT 2010 statement^22^.

### Design

A randomized experimental trial comparing self-reported pain in persistent LBP patients following SMT applied to lumbar segments of high stiffness or low PPT.

### Participants

Patients with LBP were recruited from the Spine Centre of Southern Denmark, a regional hospital that specializes in spinal pain syndromes referred by general medical practitioners, chiropractors, consultant rheumatologists, and other in-house clinicians. Participants were identified and invited to participate using two methods: (i) details of the project were included in the information sent to patients prior to their first appointment. (ii) Verbally at the clinical consultation.

All potential participants were diagnosed with persistent LBP by the clinician in charge before enrollment screening was conducted.

Inclusion criteria for the study were as follows: (a) persistent LBP for more than 3 months, without prior spinal surgery. (b) LBP of benign origin e.g. no malignancy or axial spondyloarthropathy. (c) Between 18 and 60 years of age. Exclusion criteria were (a) indications for surgical evaluation due to low back pain with/without leg pain. (b) History of SMT in the preceding 4 weeks. (c) Opioid use exceeding 40 mg of morphine or equivalent (oral intake) at the time of inclusion. (d) Comorbid conditions that could interfere with project methodology (e.g. BMI exceeding 35 and pregnancy). Further, exclusion for analysis were defined as: (a) failure to complete a minimum of 75% of the allocated intervention. (b) Received SMT or mobilization techniques to the lower back in other settings during the study. (c) Changes in pain medication during the study. The assessor in the study (CGN) recorded these parameters at each scheduled contact with the participant.

### Study protocol

An overview of the study protocol is reported here, and an extended explanation for each point of interest is provided in the *data collection* section.

#### The baseline lab session

This session in the laboratory consisted of the following: (i) completion of the patient reported outcomes, (ii) segmental markings, (iii) VT testing, (iv) PPT testing and (v) the segmental randomization.

#### The initial SMT session

The initial SMT session immediately followed the baseline lab session.

#### SMT session two to four

The three additional SMT sessions, identical to the initial SMT session, were completed over the next 14 days.

#### The Post-SMT lab session

Immediately after the fourth and final SMT session, the participant repeated the items performed in the baseline lab session (i–iv).

#### The Follow-up lab session

Fourteen (14) days after the post-SMT lab sessions, the participant repeated the items performed in the baseline lab session (i–iv).

### Data collection

#### Demographic data

Associated demographic data from each participant were extracted from the Danish SpineData questionnaire^23^ with consent.

#### Pain intensity

Patient reported LBP intensity was captured by the validated Low Back Pain Rating Scale^24^. It consists of an 11-point numerical rating scale (NRS) of *current LBP*, *average* and *worst LBP* during the last 14 days.

#### Segmental markings

Each participant was placed in a prone position on a standard examination table and the spine process of T12-S1 were identified using ultrasonography (Sonosite Titan Linear, L38 probe)^25^. The participants were instructed not to wash off skin markings during the study period. This procedure was repeated at each lab visit and skin markings refreshed.

#### VerteTrack

The VT rolls a weighted indenter along the lumbar spine of a prone subject. The resulting vertical displacement in spinal tissues is measured continuously by a string potentiometer (TE Connectivity, USA). From this, tissue stiffness (N/mm) can be determined (applied mass/displacement) along the length of the lumbar spine. The trajectory of the roller follows pre-defined skin markings through a laser mounted guidance system to obtain stiffness values. The process is repeated with discrete incrementally increasing of 1 kg up to 6 kg with a sampling rate of 30 Hz.

The resulting data were graphically smoothed using a polynomial function, and visualized using LabView version 15.0f3 for windows 10, National Instruments, Texas, USA before being extracted to a spreadsheet (LibreOffice, vers. 6.0.7.3, for Ubuntu 18.04) for further analysis. Global Stiffness (GS) was calculated as the average of the slope with the first and terminal data points removed. The comfort and safety of VT has been evaluated previously^26^ as has its reliability in an asymptomatic population^14^.

#### Pressure algometry

Pressure pain threshold was measured with the participant in the prone position, at each segment from L1 to L5 using a pressure algometer (model 2, Somedic, Hørby, Sweden). Attached to the probe was a custom, 3D printed double-headed contact (2 × 1 cm^2^, 3 cms apart), that allowed for a bilateral pressure to be applied to the skin surface at each side of the mid-line. The instrument was applied manually with a nominal rate of 50 kPa/s. A trial procedure consisting of 1–2 PPT tests were completed on the lower extremity and T12 to familiarize the participant with the procedure before spinal testing.

The PPT of each lumbar segment was measured three times with approximately 10 s rest intervals. If no pain has been elicited by 1,000 kPa, this was recorded as the PPT. If the first and second measurements were 1,000 kPa, a third would not be performed. All segments were tested in the same predetermined random order for each participant at each time point. Pressure pain threshold has excellent intra-rater reliability in a LBP cohort^27^.

See Table 1 for an overview of collected variables.

**Table 1:** An overview of the variables of interest for the analysis.

Variable name	Variable type	Data type	Description/transformation
Patient reported low back pain intensity/numerical rating scale [NRS]	Primary outcome	Continuous data [0:10]	The mean value of each NRS score in the low back pain rating scale (current, average and worst low back pain intensity)
Lumbar stiffness/global stiffness [GS]	Secondary outcome	Continuous data [0–∞]	The average slope of the force–displacement curve from the second lowest load to the second highest load allowed by protocol (~ 83 N) measured at each segment. For the analyses a mean sum score for all segments were applied
Pain sensitivity/pressure pain threshold [PPT]	Secondary outcome	Continuous data [0:1,000]	A mean score of the 3 trials (kPa) measured at each level, and for the analysis a mean sum score for all segments were applied

### Segment randomization procedure

The maximal force–displacement value (FD) from the VTs at the maximally applied load was used as an indicator of segmental stiffness, and the mean value of the three PPT measurements was used as an indicator of segmental pain sensitivity. The maximum VT displacement (FD) was used for the randomization to mimic a clinical examination. As the absolute maximum of these two parameters potentially could overlap, the identification of the ‘most stiff’ and ‘most sensitive’ segment was codified using a ratio that scored all segments between − 1 and + 1 (Eq. 1), where − 1 indicated the segment as characterized by the relatively highest degree of stiffness and lowest degree of pain sensitivity, while + 1 indicate the segment as characterized by the relatively highest degree of pain sensitivity and lowest degree of stiffness. The following algorithm was used to determine the ratio:

1 $${Segment}_{NormalizedDifference}=\frac{\left({segment}_{FD}-{min}_{FD}\right)}{\left({max}_{FD}-{min}_{FD}\right)}-\frac{\left({segment}_{PPT}-{min}_{PPT}\right)}{\left({max}_{PPT}-{min}_{PPT}\right)}$$

The absolute lowest (− 1) and highest (+ 1) ratio score were chosen as the stiffest and the most pain sensitive segment, respectively. If the resulting segments were adjacent, the remaining ratio scores were scrutinized, and if another segment had a ratio score that differed by a ratio score of less than 0.1 compared to the ratio of the absolute ‘*most stiff*’ or ‘*most sensitive*’ segment, this segment was used for the allocation instead. If no such segment existed the two original adjacent segments were chosen for allocation.

For randomization, a computer-generated list was constructed, stratified in a list of A (indicating the *stiff group*) or B’s (indicating the *pain group*) in a near 1:1 order for a total of 155 A or B characters corresponding to the maximum number of participants possible to include. The list further included a column indicating each ID number. In this fashion, each participant was given an a-priori assignment of either A or B indicating the group/segmental allocation.

### Blinding

From baseline lab data, the assessor (CGN) identified the two specific spinal segments (L1–L5) that were the most *stiff* (segment A) and had the lowest pressure *pain* threshold (segment B). The assessor was thus aware of the meaning of segments ‘A’ and ‘B’, but was blinded to the randomization list (participant ID and A/B allocation). Conversely, the treating chiropractor used the randomization list to determine the specific spinal segment to treat, but was blinded to the meaning of ‘A’ and ‘B’ as *stiff* and *pain* group. The participant was blinded to both.

### Spinal manipulative therapy

The SMT was provided in a standardized manner where the participant was placed in a side-lying position and the subsequent SMT was delivered with a high velocity, low amplitude technique^28^ targeting the randomized segment^29,30^ using a contact point at the spine process^31^. The direction of the SMT was applied in a posterior to anterior direction^32^.

Up to 3 SMT attempts were allowed for a successful treatment. Whether the treatment was successful was determined by the chiropractor and independent of the common cavitation sounds that can accompany SMT^28^. Any adverse events that occurred were recorded for each SMT session.

Two chiropractors each with more than 12 years of clinical experience performed all SMT in this study. They were instructed not to discuss the project with the participants in order to avoid influencing their assessment of treatment outcomes. No further training was completed before initiating the trial^33^.

No changes were made to the methodology after commencement of the trial.

### Statistical considerations

#### Power

A power calculation based on a 10% mean group difference in patient reported low back pain intensity [numerical scale from 0 to 10] between the *stiff* and the *pain* group with an 80% beta and a 5% alpha level indicated group sizes of 62. The 10% group difference was chosen a-priori as we did not expect a large between group mean difference^34^.

#### Descriptive data

Descriptive data is reported as means and standard deviations for normal distributed data, medians and interquartile ranges for non-normal distributed data or count and frequency for categorical data.

#### Stiffness data

Before extracting stiffness data a subjective analysis was completed in LabView. Some loads were affected by such factors as participant breathing, muscle guarding or technical errors and were subsequently removed from analysis.

#### Non-overlapping segment analysis

Both GS and PPT were obtained for each lumbar segment at three different time points (baseline, post-SMT and follow-up). As the location of these segments were repeated at each time point, it was possible that the trajectory used was not the same, this means that what was measured as L5 at baseline could have been measured as L4 at post-SMT. We inspected all the sagittal curvatures projecting the lordosis of the participants lower back, using the graphs presented by LabView and determined if the curvatures were comparable to the baseline curvature for each time point. If not, this time point was omitted for the mixed models concerning the outcomes of GS and PPT.

#### Repeated outcomes

Linear mixed models for the different outcome measures were used for the analyses, with group and time as fixed interacting effects, and subject as a random intercept using an unstructured variance–covariance all models. Model assumptions were tested for normal distribution of the residuals error using Q-Q plots and the homogeneity of variance was tested plotting the residuals vs the predicted values. All repeated outcomes are presented as mean baseline changes within group and mean differences between groups, along with 95% confidence interval and p-values, as well as a visual presentation of the mean scores and the standard errors.

A p-value < 0.05 was considered statistically significant.

Data were analyzed using R for linux (v. 3.6.0 with R-studio v. 1.1.456 and relevant add on packages from the Tidyverse^35^).

The statistical analysis plan was completed in collaboration with a biostatistician at the University of Southern Denmark.

## Results

### Participants

**Figure 1:**
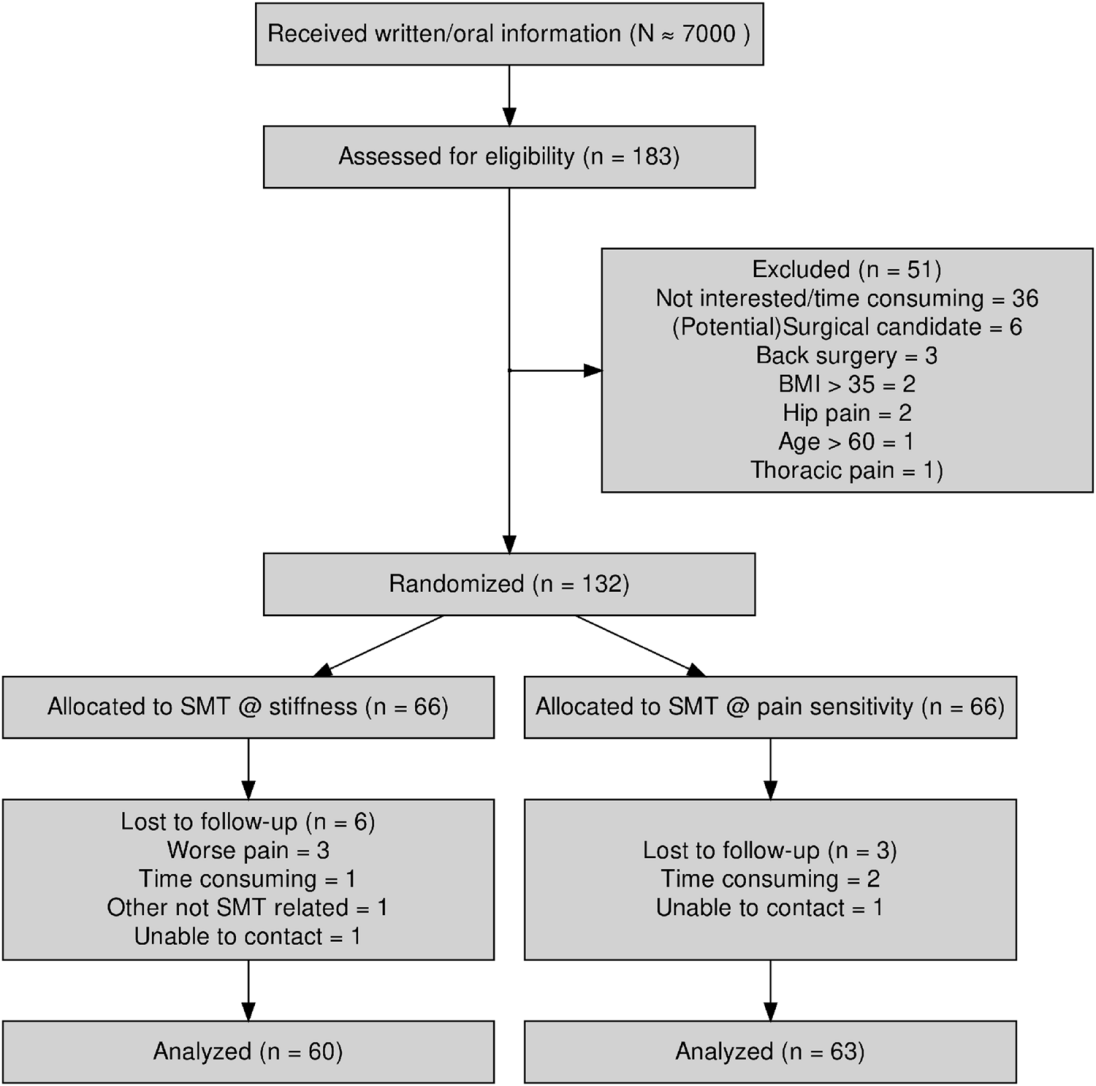
A CONSORT flow diagram of the participants enrollment, segment allocation and availability for follow-up and analysis. *SMT* spinal manipulative therapy.

A total of 132 participants were enrolled in the study between November 2017 and January 2019. Of those 132, 7 did not complete the 2 week SMT intervention, and an additional 2 were unreachable for follow-up. The result was 123 participants completed the study. No participants were excluded after initiating the trial on the basis of the exclusion criteria. See Fig. 1 for a flowchart visualizing the participant inclusion.

### Descriptive data

Table 2 shows a descriptive summary of the participant characteristics. The mean NRS score for the participants was 6 (SD = 2). The baseline GS was 4 (SD = 1) and the median value of PPT was 471 (IQR = 356). The participants were equally divided with 66 participants in each group.

**Table 2:** An overview of baseline characteristics for participants with persistent low back pain who entered a randomized experimental trial.

	Pain group, N = 66	Stiff group, N = 66
Patient reported low back pain intensity	5.64 (1.79)	5.55 (1.93)
Disability	27.42 (11.54)	28.19 (11.85)
Global stiffness	4.26 (0.75)	4.02 (0.86)
Pressure pain threshold	488.73 [330.95]	436.6 [364.9]
Age	46.74 (8.66)	43.47 (10.46)
Sex, male (%)	40 (61)	32 (48)
Low back pain duration (months)	14.10 [67.10]	17.50 [49.35]
Patient reported leg pain intensity	4.31 (2.67)	3.87 (2.77)
Overall progress since pain debut, worse (%)	34 (52)	38 (58)

Presented as mean (standard deviation), median [interquartile range] or categorical.

Albeit the present cohort consisted of fewer women, it is comparable to the general patient population of our unit in terms of age, pain duration and intensity^23^.

### Non-overlapping segment analysis

The analysis concerning non-overlapping segments for repeated spinal measures resulted in the exclusion of 11.7% of the GS and PPT measures at different time points. These data points were omitted and not used in the final analysis. Furthermore, one baseline GS trial was faulty and therefore, omitted. As a result, the total data size (each subject measured three times) was reduced from 369 data points to 325 data points.

### Spinal manipulative therapy

All four sessions were completed by 119 participants while 7 completed three. This was not adjusted for in the analysis. The average duration of the intervention period was 13 (SD = 6) days and follow-up occurred 14 (SD = 6) days afterwards.

#### Adverse events

Of the participants who completed the intervention, 69% reported minor side effects. This included a reporting of increased local muscle pain by 63%, 33% reported increased lumbar stiffness, 10% reported headaches and 8% reported worsening of leg pain, only 5% had other minor side effects such as nausea, dizziness etc. One participant reported continued anterior chest pain after the 4th session at follow-up.

### Linear mixed model for group comparison over time

All models assumptions were upheld. See Table 3 for a summary of within mean changes and between group mean differences for the three outcome measures (i) NRS, (ii) GS and (iii) PPT.

**Table 3:** Changes in patient reported low back pain intensity, lumbar stiffness and pressure pain threshold in participants with persistent low back pain who are treated with spinal manipulative therapy over 4 sessions at either a *pain segment* or a *stiff segment*.

Within group					Between group	
Time	Within group mean change, estimate (95% CI)		p-value		Between group mean difference, estimate (95% CI)	p-value
	Pain	Stiffness	Pain	Stiffness	Pain–stiffness	Pain–stiffness
**Patient reported low back pain intensity (numerical pain rating scale)**						
Baseline to post-SMT	− 0.70 (− 1.12 to − 0.28)	− 0.60 (− 1.03 to − 0.17)	< 0.001	< 0.001	0.11 (− 0.49 to 0.71)	0.68
Baseline to follow-up	− 0.66 (− 1.08 to − 0.24)	− 0.77 (− 1.20 to − 0.34)	< 0.001	< 0.001	− 0.11 (− 0.71 to 0.49)	0.67
**Low back stiffness (global stiffness, N/mm)**						
Baseline to post-SMT	0.03 (− 0.22 to 0.29)	0.04 (− 0.22 to 0.30)	0.76	0.74	0.00 (− 0.36 to 0.37)	0.98
Baseline to follow-up	0.08 (− 0.18 to 0.34)	− 0.05 (− 0.32 to 0.22)	0.48	0.66	− 0.13 (− 0.5 to 0.24)	0.42
**Low back pain mechanical pain sensitivity (pressure pain threshold, kPa)**						
Baseline to post-SMT	99.29 (56.78 to 141.8)	33.00 (− 10.71 to 76.71)	< 0.001	0.08	− 66.29 (− 127.27 to − 5.32)	0.01
Baseline to follow-up	90.02 (46.54 to 133.5)	48.51 (3.38 to 93.64)	< 0.001	0.01	− 41.51 (− 104.18 to 21.16)	0.12

Within mean changes and between group mean differences are presented as mean differences between baseline and post-SMT/follow-up and between group mean with 95% confidence intervals.

### Patient reported low back pain intensity

Both groups reported a significant decrease of NRS at post-SMT and follow-up, but there was no significant difference between the groups at any time point (group mean difference at: post-SMT of 0.11, p-value = 0.68, follow-up of 0.11, p-value = 0.67).

### Global stiffness

There was no statistically significant within mean group changes for GS nor any between group differences. On average, a decrease in GS score was observed in the *stiff group* at follow-up. Conversely, the average GS score in the *pain group* increased.

### Pressure pain threshold

The mean PPT scores increased over time for both groups. In the *pain group*, PPT increased significantly at both post-SMT and follow-up compared to baseline. The *stiff group* also demonstrated a significant increase in PPT, but only at follow-up. The *pain group* reported a significant higher mean PPT score compared to the *stiff group* of 66.29 kPa at post-SMT.

## Discussion

This is the first trial that investigated whether clinical and experimental differences would be observed when randomly directing SMT at pre-targeted segments of increased stiffness or pain. Both groups responded to treatment with an overall decrease in the primary outcome patient reported LBP intensity, but there was no statistically or clinically significant difference between groups. Likewise for the secondary outcomes, stiffness did not change significantly throughout the study but PPT increased significantly in both groups at follow-up and a large between group difference in PPT was observed directly post-SMT, indicating that PPT increased at a much larger rate directly post-SMT for the *pain* group compared to the *stiff group*. However, PPT stagnates from post-SMT to follow-up for the *pain group*.

### Patient reported low back pain intensity

Whether the reduction observed in patient reported LBP intensity is clinically significant is debatable and is overall lower compared to the majority of SMT trials on persistent LBP^21^. This result, could be explained by many different possibilities. The first possibility is that the intervention was not sufficient as the number of sessions was limited to four over 14 days; a number of treatments shown in the literature to predict overall improvement in primary care chiropractic patients^36^. The second possibility was that the participants were more likely to be complex^37^ given their recruitment from a secondary treatment facility; they may have been more likely to be non-responders to SMT. Third, a longer follow-up period could have resulted in larger improvements^21^. Last, the planned intervention was experimental in nature and limited the SMT application to a single lumbar segment with no adjunct therapy.

### Lumbar stiffness

Quantifying spinal stiffness is a relatively new field in spine research. Early experiments evaluating stiffness demonstrated a decrease in spinal stiffness in LBP patients who responded positively to SMT^38^. These results were subsequently repeated^12^ although the duration of LBP in that cohort was not described in detail. Results since then have been mixed, as different studies have used the measure to evaluate associations with other outcomes^39^ or in other parts of the spine^40^.

As such, SMT may have a differential impact on stiffness in subgroups of LBP patients. Arguably, the stiffness associated with acute or trivial LBP could be due to inflammation or muscle guarding, whereas stiffness associated with chronic or non-trivial LBP could potentially be due to more degenerative changes, intervertebral fibrosis or muscle inhibition/atrophy. This is speculative, but is supported by an exploratory analysis reporting that SMT responders tends to have a lower prevalence of degeneration and a higher degree of disc diffusion^41^. The responder status of participants receiving SMT appears to modify the changes in stiffness^12,39^. The present study, did not take the inclusion of responders into account, and possibly the lack of stiffness change was due to the chronicity of cohort and the minor changes in patient reported LBP intensity.

### Pain sensitivity

The literature concerning the hypoalgesic effect of SMT is conflicting^15–17^. This may be the result of differences in study populations as they often are heterogeneous, and includes asymptomatics participants^17^ as well as participants with musculoskeletal conditions often found in the general public or primary care^16^. Further, these studies are experimental in nature, often using a single SMT session, followed by an immediate PPT reassessment^15–17^.

The mechanism behind a hypoalgesic effect of SMT is unclear and two possible explanations exists: (i) SMT could have a neurologically mediated reflex independent of clinical improvement that would give rise to an immediate change in pain sensitivity or (ii) SMT has a curative effect on a mechanical relationship/segmental dysfunction. This in itself affects pain sensitivity, which arguably would give rise to a more profound and longer lasting effect on pain sensitivity. Multiple studies have measured different QST measurements before and after SMT at a “manipulative lesion” each finding an immediate decrease in the QST measurement^42–45^. However, when pooling the results in a systematic review, there was no greater hypoalgesic effect compared to a predetermined location^15^. An obvious weakness in these studies is the reliability of locating the “manipulative lesions”.

Manual palpation has limited value in clinical practice, and appears to have no impact in modifying the hypoalgesic effect of a single SMT session^15^. As discussed, the hypoalgesic effect of SMT may be related to a presumed curative effect on underlying segmental dysfunctions which likely would require multiple sessions at the affected segment. In this scenario, the PPT change could progress gradually over time rather than immediately post-treatment. A recent two-armed trial^46^ directed SMT at lumbopelvic region predetermined beforehand over multiple sessions in a chronic LBP population, a significant difference within group was found but none when compared to sham. This supports our finding that PPT change following SMT is a neurologically mediated reflex that is segment dependent (e.g. a segment with low PPT).

### Neurological mediated effect of SMT

This neurological mediated reflex could depend upon sensitization of central pain mechanisms. This mechanism occurs as the pain persists^47^ and typically the QST scores differs significantly in chronic versus acute pain^48^ and versus asymptomatic subjects^49^. There is some evidence to suggest that such widespread hypersensitivity is rapidly reversible^50,51^. It is possible, albeit speculative that central hypersensitivity has to occur before a robust hypoalgesic effect of SMT can be observed.

Interestingly, the observed increase in PPT was not a proxy for clinical improvement of subjective pain relief. This suggests that multiple factors are important for locating the relevant segmental dysfunction or that multiple segments are responsible; SMT application should not be limited to pain provocation or stiffness assessment alone^52^.

### Limitations

The random allocation makes it possible to explore the effect between the 2 groups. All the tests were completed by one assessor thus eliminating inter-rater variability. Furthermore, the allocation was blinded for all involved. As we did not compare with a sham-SMT treatment, the present study does not shed light on whether SMT was responsible for the changes observed in outcome measures. It is possible that any mechanical sensory input could provide the same results. However, investigating the causality of SMT was not the overall purpose of the paper.

Although this was a randomized trial, numerous non-systematic errors could occur with respect to repeated SMT to the assigned segment. The present study tried to minimize this risk by using ultrasonography and skin-surface marking of vertebral location. Ultrasonography was, however, only completed at each lab visit, and some markings disappeared between visits meaning that static palpation was used to locate the indicated segment. The thrust used in SMT, albeit, directed at one segment, can result in cavitation on multiple segments^53^ which further decreases the specificity of SMT application. However, this is the case for all studies investigating any manual therapy and cannot be controlled.

A significant and possible modifying factor was the randomization process. There was no prior research that had compared stiffness and pain sensitivity, Therefore, the study used absolute values for pain and stiffness. It is not evident whether the randomization actually represents the stiffest or most pain sensitive segments. It may be possible that a discrete anatomical distribution of lumbar stiffness exists that ought to be adjusted for, yet for PPT this does not appear to be the case^49^. The indexing was used to avoid a potential overlap between the stiffest and most pain sensitive segment. A pilot study was completed, before initiating the current study, that included 20 participants with persistent LBP and the overlap was approximately 25% (data unpublished).

In this study, the measurement techniques as well as the applied SMT likely differ from procedures used in clinical practice. Many techniques used here, although objectively quantifiable, are unidirectional, while both palpation and manual pressure can be directed at multidirectional planes. Further, the randomization procedure that guided the treatment in this study was only performed at baseline, it is possible that the ratio between stiffness and pain could change during the course of the experiment.

In this present study, no stratified analysis on responder/non-responder status was performed. It is possible, that such analysis could demonstrate important differences in secondary outcome measures between those with and without clinical improvement following SMT. This is the subject of a secondary analysis and future publication. Similarly, between-group differences in the secondary outcome measure (disability) is not presented here.

Finally, the primary objective of our study was patient reported LBP intensity and the sample size was calculated to be respondent for changes in this parameter. This leaves the possibility that the analyses of the secondary objectives were under powered. Further, some of the repeated data were omitted due to the non-overlapping spine trajectories, further increasing the risk of the analyses being under powered.

## Conclusion

No difference between SMT applied to the most *stiff* vertebra or the most *painful* vertebra were found to improve the primary outcome, patient reported low back pain intensity or the secondary outcome, spinal stiffness. However, a large difference in the secondary outcome, pressure pain threshold was observed post-SMT.

This suggests that in the patient population studied, SMT appears to impact pain sensitivity in a specific segmental fashion and the effect is mediated by a neurological reflectory system. By comparison, the mechanical measure of spinal stiffness was not affected by the application site.

## Data Availability

Data is available upon request, please contact casper.nim@rsyd.dk.
